# Age- and BMI-stratified assessment of serum anti-Müllerian hormone as a biomarker for polycystic ovary syndrome diagnosis in Chinese women

**DOI:** 10.1186/s12902-025-02136-3

**Published:** 2026-01-16

**Authors:** Yang You, Zaixin Guo, Xinyu Hong, Xiaohui Li, Shuwen Chen, Meng Xiao, Xuesong Shang, Xinqi Cheng, Meizhi Liu, Fang Zhao, Rui Li, Qi Yu

**Affiliations:** 1 Department of Obstetrics and Gynecology, National Clinical Research Center for Obstetric & Gynecologic Diseases, Peking Union Medical College Hospital, Chinese Academy of Medical Sciences & Peking Union Medical College, Peking Union Medical College Hospital (Dongdan Campus), No. 1 Shuaifuyuan Wangfujing Dongcheng District, Beijing, 100730 China; 2 Department of Laboratory Medicine, Peking Union Medical College Hospital, Chinese Academy of Medical Sciences & Peking Union Medical College, Peking Union Medical College Hospital, Beijing, 100730 China

**Keywords:** Polycystic ovary syndrome, Anti-mullerian hormone, Age, Body mass index, Diagnosis, Infertility

## Abstract

**Background:**

This study aimed to establish the diagnostic cut-off value of serum Anti-Müllerian Hormone (AMH) for Polycystic Ovary Syndrome (PCOS) in Chinese women of reproductive age and explore its diagnostic efficacy, particularly in relation to age, body mass index, and PCOS phenotypes.

**Methods:**

A prospective case-control study was conducted in 264 PCOS patients and 190 healthy controls from December 2021 to December 2023. Serum AMH levels were measured, and Receiver Operating Characteristic curves were plotted to assess diagnostic efficacy. PCOS was classified into four phenotypes based on Rotterdam criteria.

**Results:**

Serum AMH levels were significantly elevated in PCOS patients compared to controls (4.29 ng/mL vs. 8.97 ng/mL, *P* < 0.001). The cut-off value of AMH for diagnosing PCOS was 6.105 ng/mL, with an area under the curve (AUC) of 0.832, sensitivity of 0.739, and specificity of 0.768. Diagnostic performance varied across PCOS phenotypes, with the highest AUC observed in Phenotype A (0.865). AMH combined with other sex hormones improved diagnostic efficacy (AUC = 0.923). AMH correlated positively with ovarian volume, LH, and testosterone but negatively with age, fasting insulin, 2-hour postprandial insulin, and HOMA-IR. AMH was an independent risk factor for infertility in PCOS patients (OR = 1.058).

**Conclusions:**

Serum AMH is a valuable diagnostic marker for PCOS in Chinese women of reproductive age, with phenotype-specific diagnostic cut-offs. AMH combined with other sex hormones enhances diagnostic accuracy. Age and BMI influence AMH cut-offs, and higher AMH levels are associated with more severe PCOS phenotypes and increased infertility risk.

**Clinical trial number:**

Not applicable.

**Supplementary Information:**

The online version contains supplementary material available at 10.1186/s12902-025-02136-3.

## Background

Polycystic ovary syndrome (PCOS) is one of the most common endocrine disorders affecting women of reproductive age, affecting approximately 8%-13% of women of reproductive age worldwide [1]. An epidemiological survey targeting Chinese women has revealed a significant increase in PCOS prevalence within the 20-44-year age group, rising from 5.6% in 2010 to 8.6% in 2020, reflecting a 66% increase over the decade [2]. Its diagnosis is based on the 2003 Rotterdam criteria, which necessitate the presence of at least two of the following three features: oligo- or anovulation, clinical or biochemical signs of hyperandrogenism, and polycystic ovarian morphology (PCOM) detected by ultrasound, while excluding other related endocrine disorders [3]. Patients with PCOS exhibit a heightened risk of developing infertility, miscarriage, obesity, cardiovascular and cerebrovascular diseases, asthma, as well as mental and psychological disorders, highlighting the paramount significance of early diagnosis and prompt treatment for PCOS [4].

Ultrasonographic diagnosis of PCOM traditionally relies on ovarian volume and follicle number per ovary (FNPO), with established criteria defining PCOM as the presence of either ≥ 12 follicles measuring 2–9 mm in diameter or an ovarian volume exceeding 10 mL in at least one ovary [3]. Prior to the international PCOS guidelines, the diagnostic basis for PCOM lacked corresponding evidence-based medical evidence. However, the diagnosis of FNPO relies on the sensitivity of the examiner and the equipment, with the FNPO count increasing with improved equipment sensitivity [5]. The latest international PCOS guideline recommends the definition of PCOM in adults as meeting at least one of three ultrasonographic parameters in one or both ovaries: FNPO ≥ 20, ovarian volume ≥ 10 ml, or follicle number per cross-section (FNPS) ≥ 10 [6]. Additionally, ultrasonic examination involves significant inter-center variability in measurements, as demonstrated by variations in both antral follicle count and AMH levels across different facilities [7]. The ultrasonic detection method (transabdominal versus transvaginal) and operator experience also significantly impact the results [5, 8].

The technical limitations of ultrasonography in PCOM diagnosis, particularly its operator dependence and measurement variability, emphasize the need for more objective biomarkers [9]. Serum AMH, being a direct reflection of follicular pool size, is considered a promising alternative as it strongly correlates with antral follicle count while remaining independent of technical or operator-dependent factors [10]. The latest guideline additionally suggests that serum AMH levels can be utilized to assess PCOM in adults, albeit it is not a necessary condition for diagnosing PCOS. If menstrual irregularity coexists with hyperandrogenism, further ultrasound or AMH level testing is not required [6]. However, in adolescent populations, the guideline explicitly discourages AMH use for PCOS diagnosis due to physiological AMH elevation during pubertal development and insufficient diagnostic accuracy evidence [6].

AMH is a dimeric glycoprotein hormone belonging to the transforming growth factor (TGF-β) family [11]. Within the female reproductive system, the granulosa cells of preantral and small antral follicles secrete AMH, which exerts inhibitory effects on primordial follicle recruitment and diminishes follicular sensitivity to follicle stimulating hormone (FSH) [11, 12]. Numerous studies have consistently shown that women with PCOS demonstrate significantly higher serum AMH levels relative to their healthy counterparts [13–15]. AMH may participate in the pathogenesis of PCOS by affecting follicular development and maturation disorders, inhibiting follicular aromatase activity, exacerbating hyperandrogenism, and mediating central neuroendocrine disorders in PCOS [16].

Despite its potential utility, the clinical application of AMH for PCOS diagnosis encounters several significant challenges that require careful consideration. The establishment of reliable diagnostic thresholds remains problematic, as evidenced by the wide range of proposed cutoff values (1.40–7.98 ng/ml) and variable predictive accuracy (area under the curve (AUC) 0.66–0.994) reported across different populations [5]. Among Asian women aged 20–43 years, a prospective cohort study established an optimal AMH cutoff of 4.0 ng/ml, demonstrating reasonable diagnostic accuracy with an AUC of 0.81, sensitivity of 0.72, and specificity of 0.76 [17]. These findings are supported by a comprehensive meta-analysis of 62 adult PCOS studies, which reported pooled sensitivity and specificity estimates of 0.79 (0.76–0.82) and 0.87 (0.84–0.89), respectively, for AMH in PCOS diagnosis [18]. These discrepancies stem from population-specific factors such as age, ethnicity, and BMI, which significantly influence AMH levels and diagnostic performance [6]. Additionally, methodological differences across detection platforms further contribute to variability in results [6]. Although current guidelines recognize AMH as an alternative to ultrasound for assessing PCOM, they emphasize the need for locally validated thresholds rather than universal cutoffs, reflecting the importance of accounting for demographic and technical heterogeneity in clinical implementation [6].

To address these gaps, we conducted a prospective case-control study in Chinese women of reproductive age to establish clinically relevant AMH thresholds for PCOS diagnosis using ROC analysis. Given that both age and BMI are known to significantly influence AMH levels, with AMH showing a progressive decline with advancing age and potentially being affected by obesity-related metabolic alterations, we specifically designed our study to explore diagnostic thresholds and efficacy of AMH across different age and BMI groups [12, 19–22]. This stratified approach allows for more precise clinical application of AMH cut-off values, accounting for the physiological variations in ovarian reserve associated with age and BMI.

Existing evidence supports AMH’s diagnostic utility for PCOS, yet establishing appropriate reference values requires careful consideration of population-specific factors. This study provides clinically relevant data by determining age- and BMI-stratified AMH thresholds for Chinese women, while also assessing the diagnostic performance of AMH in combination with other hormonal parameters. Our findings offer practical guidance for PCOS diagnosis in clinical settings where ultrasound evaluation presents limitations, particularly for the Chinese population where such reference data were previously lacking.

## Methods

### Study design and participants

This study is a single-center, prospective, case-control study conducted at the Gynecological Endocrinology/Reproductive Center of Peking Union Medical College Hospital (PUMCH) from December 2021 to December 2023. This study employed a prospective case-control design to rigorously compare AMH levels between carefully phenotyped PCOS patients and matched healthy controls, a fundamental requirement for establishing valid diagnostic thresholds. The prospective nature of the study facilitated standardized collection of comprehensive clinical, hormonal and metabolic data, while the single-center design maintained strict consistency in diagnostic criteria implementation, laboratory methodologies, and ultrasonographic assessments throughout the investigation period. The study was approved by the Institutional Review Board (IRB) of PUMCH (No. HS-3255).

The primary endpoint was to determine the optimal diagnostic cutoff value of serum AMH for PCOS in reproductive-aged Chinese women by constructing receiver operating characteristic (ROC) curves comparing PCOS patients with healthy controls.

The secondary endpoints focused on evaluating AMH’s combined diagnostic utility with key hormonal markers (luteinizing hormone (LH), LH/FSH ratio, FSH, and total testosterone (TT)), characterizing its correlations with clinical and metabolic parameters, assessing its role as an infertility risk factor in PCOS, and establishing age- and BMI-stratified reference ranges to improve subgroup-specific diagnostic accuracy.

Based on the hypothesis that AMH can effectively distinguish between PCOS and healthy controls, the required sample size was estimated using PASS software. Ultimately, 264 PCOS patients and 190 healthy controls were recruited, all of whom provided written informed consent.

Eligibility for the PCOS group required women aged 18–40 years to fulfill at least two of the following: (1) oligo- or anovulation (OA), specified as menstrual cycle length > 35 days or ≤ 8 menstrual periods annually; (2) clinical hyperandrogenism (HA, a modified Ferriman-Gallwey (mF-G) score ≥ 4) or biochemical hyperandrogenism (total testosterone ≥ 0.41 ng/mL); and (3) PCOM confirmed by transvaginal/ transrectal ultrasound. Exclusion criteria included congenital adrenal hyperplasia, Cushing’s syndrome, androgen-secreting tumors, and oral contraceptive use within 3 months [3, 23].

Following the Rotterdam criteria [3], the PCOS group was further classified into four phenotypes: Phenotype A (OA + HA + PCOM), Phenotype B (OA + HA), Phenotype C (HA + PCOM), and Phenotype D (OA + PCOM).

The healthy control group consisted of women aged 18–40 years meeting all the following criteria: (1) eumenorrheic (21- to 35-day cycles); (2) absence of clinical and biochemical hyperandrogenism; and (3) sonographic absence of PCOM. Exclusion criteria included BMI ≥ 40 kg/m², oral contraceptives use within 3 months, pregnancy, ovarian abnormalities, endocrine or metabolic diseases, and malignant tumors.

Participants were stratified by age into four groups: 18–25 years, 26–30 years, 31–35 years, and 36–40 years [19]. The age stratification was carefully selected based on established clinical and biological considerations. These groupings reflect clinically meaningful reproductive stages, capturing the well-documented decline in ovarian reserve and fertility potential that becomes particularly pronounced after age 30–35 [24, 25]. From a biological perspective, this categorization aligns with the characteristic trajectory of AMH levels, which typically peak around age 25 before entering a gradual decline phase [12]. Furthermore, this approach maintains consistency with validated methodologies employed in previous AMH studies, which have demonstrated the sensitivity of these age intervals for detecting clinically relevant differences in ovarian function [19, 26].

Participants were categorized into an underweight/normal weight group (BMI < 25 kg/m²) and an overweight/obese group (BMI ≥ 25 kg/m²) [27].

Infertility was defined as the inability to achieve a clinical pregnancy following ≥ 12 months of regular unprotected sexual intercourse among couples of reproductive age [28]. While our primary focus was PCOS-related ovulatory dysfunction, we did not exclude cases with concurrent male factor infertility or tubal factors to better reflect real-world clinical populations.

### Data collection

All participants underwent comprehensive baseline evaluations during the early follicular phase (days 2–4 of spontaneous or progestin-induced menstrual cycles). Clinical assessments included menstrual, reproductive and gynecological history, physical examination with anthropometric measurements (height, weight, waist and hip circumference), and standardized evaluation of hyperandrogenic manifestations using the Comprehensive Acne Severity Scale (CASS), mF-G score for hirsutism (cutoff > 4), and Ludwig scale for alopecia [23, 29, 30].

Blood tests measured: (1) reproductive hormones - AMH, FSH, LH, TT, prolactin (PRL), sex hormone-binding globulin (SHBG); (2) metabolic parameters - fasting blood glucose (FBG), fasting insulin (FINS), 2-hour postprandial glucose (2 h-PG) and insulin (2 h-PI) after 75 g oral glucose tolerance test; and (3) lipid profile including triglycerides (TG), total cholesterol (TC), low-density lipoprotein cholesterol (LDL-C), high-density lipoprotein cholesterol (HDL-C).

Transvaginal or transrectal ultrasound was performed to evaluate ovarian morphology. PCOM was defined as an ovarian volume > 10 mL or ≥ 12 follicles (2–9 mm in diameter) in either ovary, per Rotterdam criteria [3]. Due to clinical reporting conventions, most ultrasound reports documented follicular status categorically (e.g., “follicles ≥ 12”) rather than providing exact FNPO counts. While quantitative FNPO data were not routinely recorded in clinical reports, the presence of PCOM was consistently documented based on these criteria.

Standard formulae were applied for key calculations as detailed in Supplementary Table 3. This includes equations for ovarian volume, BMI, waist to hip ratio (WHR), free androgen index (FAI), and homeostasis model assessment of insulin resistance (HOMA-IR), presented with their respective components and units to facilitate reference.

Serum AMH levels were measured using the automated DxI800 chemiluminescence immunoassay system (Beckman Coulter, Inc.) with manufacturer-matched reagents. The assay demonstrates a measurable range of 0.74–16.06 ng/mL. All other laboratory parameters were measured by the Department of Laboratory Medicine at PUMCH. To ensure analytical reliability, our laboratory maintained rigorous quality control through regular calibration with manufacturer-provided standards and participation in external quality assessment programs.

### Statistical analyses

Data were organized and analyzed using SPSS software version 25.0 (IBM Corp., Armonk, NY, USA).

The normality of continuous variables was evaluated using the Kolmogorov-Smirnov test. Continuous variables adhering to normal distribution were summarized using mean ± standard deviation, whereas those deviating from normality were presented with median and interquartile range (IQR). Categorical variables were described using frequency and percentage.

For comparisons between two groups, the Mann-Whitney U test and the chi-square test were utilized to analyze continuous and categorical variables, respectively. When comparing multiple groups, the Kruskal-Wallis test was employed to evaluate differences in non-normally distributed continuous variables or those failing to meet the assumption of variance homogeneity, with subsequent pairwise comparisons conducted.

Spearman’s correlation analysis was conducted to evaluate correlations between continuous variables that were not normally distributed, with the correlation coefficient r indicating the strength of the relationship between variables.

ROC curves were utilized to assess the discriminatory power of different indicators. Diagnostic efficacy was compared based on the AUC, sensitivity, specificity, and Youden index. The optimal cut-off value, associated with the highest Youden index (calculated as sensitivity + specificity − 1), was identified alongside its corresponding sensitivity and specificity [26, 31, 32].

Multivariate logistic regression analysis was conducted to investigate the influence of AMH on infertility among PCOS patients, accounting for potential confounding variables. The findings were reported using odds ratios (OR) with corresponding confidence intervals (CI), and statistical significance was determined at *P* < 0.05.

All statistical figures, including ROC curves and scatter plots comparing AMH levels between groups, were generated using GraphPad Prism 8.0 software (GraphPad Software, San Diego, CA, USA). These visualizations encompassed diagnostic performance analyses and subgroup comparisons stratified by age and BMI categories.

## Results

### Baseline characteristics of the control group and the PCOS group

This study enrolled 190 healthy controls and 264 patients with PCOS. We conducted a comparative analysis of baseline characteristics between the two groups (Table 1). The results revealed no statistically significant differences in age (*P* = 0.157) and age at menarche (*P* = 0.152) between the control and PCOS groups.

**Table 1:** Baseline characteristics of the control group and the PCOS group

Characteristics	Control(*n* = 190)	PCOS(*n* = 264)	*P*-value
	Median (IQR) OR *N*(Percentage)	Median (IQR) OR *N*(Percentage)	
Age(years)	27 (24, 32)	27 (23, 30)	0.157
BMI (kg/m^2^)	21.2(19.5, 23.2)	23.0(20.7, 26.4)	<0.001
BMI category			<0.001
BMI < 25 kg/m^2^	174(91.6%)	173(65.5%)	
BMI ≥ 25 kg/m^2^	16(8.4%)	91(34.5%)	
WHR	0.76(0.74, 0.80)	0.81(0.77, 0.85)	<0.001
mF-G score	1(0, 3)	3(2, 6)	<0.001
Facial acne	0(0, 1)	1(0, 2)	<0.001
Chest acne	0(0, 0)	0(0, 1)	<0.001
Back acne	0(0, 1)	0(0, 1)	0.020
Alopecia	0(0, 0)	1(0, 1)	<0.001
Ovarian volume (cm3)	5.27(3.74, 6.61)	9.00(7.43, 11.84)	<0.001
Infertility			<0.001
YES	0 (0.0%)	58 (22.0%)	
NO	190 (100.0%)	206 (78.0%)	
Age at Menarche (years)	13(12, 14)	13(12, 14)	0.129
Times of menstrual Cycle	12(12, 12)	7(4, 10)	<0.001
Menstrual duration (day)	5(5,7)	6(5,7)	0.012
Menorrhagia			0.009
YES	19 (10.0%)	50 (18.9%)	
NO	171 (90.0%)	214 (81.1%)	
FSH (IU/L)	6.67 (5.47, 7.66)	6.23 (5.30, 7.19)	0.002
LH (IU/L)	4.47 (3.26, 5.84)	10.91 (6.85, 15.94)	<0.001
LH/FSH ratio	0.65 (0.46, 0.87)	1.78 (1.15, 2.56)	<0.001
TT (ng/mL)	0.47 (0.37, 0.59)	0.58 (0.44, 0.73)	<0.001
PRL (ng/mL)	17.80 (13.10, 25.63)	12.00 (8.71, 15.80)	<0.001
SHBG (nmol/L)	48.85 (35.78, 66.33)	32.90 (20.65, 52.73)	<0.001
FAI	3.2(2.1, 5.1)	6.2(3.4, 10.7)	<0.001
AMH (ng/mL)	4.29 (2.51, 5.99)	8.97 (5.94, 13.03)	<0.001
FBG (mmol/L)	4.8 (4.5, 5.1)	5.0 (4.7, 5.2)	<0.001
2 h-PG (mmol/L)	5.4 (4.8, 6.4)	6.0 (5.3, 7.0)	<0.001
FINS (uIU/mL)	7.0 (5.0, 8.9)	9.6 (6.7, 15.7)	<0.001
2 h-PI (uIU/mL)	35.3 (24.2, 54.6)	42.6 (27.8, 79.4)	<0.001
HOMA-IR	1.45 (1.07,1.95)	2.13 (1.47, 3.70)	<0.001
TG (mmol/L)	0.71 (0.51, 0.96)	0.96 (0.63, 1.34)	<0.001
TC (mmol/L)	4.35 (3.94, 4.97)	4.74 (4.23, 5.19)	<0.001
LDL-C (mmol/L)	2.43 (2.11, 2.97)	2.90 (2.44, 3.32)	<0.001
HDL-C (mmol/L)	1.43 (1.28, 1.66)	1.32 (1.14, 1.48)	<0.001

Data are presented as median (interquartile range) OR N (Percentage)

PCOS, polycystic ovary syndrome; BMI, body mass index; WHR, waist to hip ratio; mF-G: modified Ferriman-Gallwey; FSH, follicle stimulating hormone; LH, luteinizing hormone; TT, total testosterone; PRL, prolactin; SHBG, sex hormone-binding globulin; FAI, free androgen index; AMH, anti-Müllarian hormone; FBG, fasting blood glucose; 2h-PG, 2-hour postprandial blood glucose; FINS, fasting insulin; 2h-PI, 2-hour postprandial insulin; HOMA-IR, homeostasis model of assessment-insulin resistance; TG, triacylglycerol; TC, total cholesterol; LDL-C, low-density lipoprotein cholesterol; HDL-C, high-density lipoprotein cholesterol

Mann-Whitney U test is utilized for analysis the continuous variables. Categorical variables are analyzed using the chi-square test. *P*<0.05 is considered statistically significant

PCOS patients showed markedly higher anthropometric measures, including BMI and WHR, along with greater prevalence of overweight/obesity (34.5% vs. 8.4%; *P* < 0.001). Androgen-related clinical manifestations were more severe in PCOS patients, as evidenced by higher mF-G scores and increased acne and alopecia severity scores (*P* < 0.05).

Reproductive markers differed significantly between groups, with PCOS patients exhibiting larger ovarian volumes (9.00 vs. 5.27 cm³; *P* < 0.001) and higher infertility prevalence (22.0% vs. 0.0%; *P* < 0.001). Menstrual irregularities were also prominent, characterized by prolonged duration but fewer annual cycles.

Endocrine profiles showed significant differences between groups. PCOS patients had higher median LH levels, LH/FSH ratios, total testosterone (0.58 vs. 0.47 ng/mL; *P* < 0.001), and FAI values (6.2 vs. 3.2; *P* < 0.001). AMH levels were 2.1-fold higher in PCOS patients (8.97 vs. 4.29 ng/mL; *P* < 0.001).

Metabolic profiling confirmed insulin resistance in PCOS patients, demonstrating significantly elevated FBG, 2 h-PG, FINS, 2 h-PI, and HOMA-IR values compared to controls. Lipid profiles showed increased triglycerides and LDL-C, with decreased HDL-C levels.

### Baseline characteristics of the four phenotypes of PCOS

The Rotterdam criteria classify PCOS into four phenotypes with distinct clinical implications [3]. Phenotype A (classic PCOS) typically presents with the most severe metabolic and reproductive complications. These phenotypic differences directly inform prognosis and therapeutic decision-making in clinical practice. Our cohort comprised 264 PCOS patients stratified as: Phenotype A (*n* = 135), Phenotype B (*n* = 20), Phenotype C (*n* = 12), and Phenotype D (*n* = 97). The median serum AMH levels varied across phenotypes, with Phenotype A exhibiting 10.11 ng/mL, Phenotype B showing 5.75 ng/mL, Phenotype C at 6.80 ng/mL, and Phenotype D reaching 8.38 ng/mL (Table 2).

**Table 2:** Baseline characteristics of four phenotypes of PCOS

Variables	PCOS-A	PCOS-B		PCOS-C		PCOS-D	*P*-value
	(*n* = 135)	(*n* = 20)		(*n* = 12)		(*n* = 97)	
	Median (IQR) OR mean ± standard	Median (IQR) OR mean ± standard		Median (IQR) OR mean ± standard		Median (IQR) OR mean ± standard	
Age(years)	26 (23, 30)	26 (23, 31)		27 (24, 29)		29 (25, 31)	0.062
BMI (kg/m^2^)	22.9 (20.7, 26.4)	22.0 (19.6, 23.6)		23.6 (19.8, 27.8)		23.3 (21.2, 26.3)	0.348
WHR	0.81 (0.77, 0.85)	0.79 (0.76, 0.81)		0.81 (0.73, 0.84)		0.82 (0.78, 0.85)	0.207
mF-G score	5 (2, 8) ^**d**^	7 (5, 11) ^**d**^		6 (4, 8) ^**d**^		2 (1, 3) ^**a, b,c**^	<0.001
Facial acne	1(1, 2)	2(0, 2)		2(1, 3)		1(0, 2)	0.626
Chest acne	0(0, 1)	0(0, 1)		0(0, 0)		0(0, 1)	0.228
Back acne	0(0, 1)	1(0, 1)		0(0, 0)		0(0, 1)	0.067
Alopecia	1(0, 1)	1(0, 1) ^**c**^		0(0, 0) ^**b**^		0(0, 1)	0.042
ovarian volume (cm3)	9.43 (7.97, 13.10) ^**b**^	6.68 (5.50, 7.99) ^**a, d**^		8.89 (7.47, 11.67)		9.00 (7.21, 11.44) ^**b**^	<0.001
Infertility	30(22.2%)	3(15.0%)		1(8.3%)		24(24.7%)	0.606
Age at Menarche (years)	13(12, 13)	13(12, 14)		13(11, 13)		13(12, 14)	0.029
Times of menstrual cycle	6 (4, 10) ^**c**^	8 (4, 10) ^**c**^		12 (11, 12) ^**a, b,d**^		8 (5, 10) ^**c**^	<0.001
Menstrual duration (day)	6 (5, 7)	6 (4, 6)		6 (5, 7)		6 (5, 7)	0.228
Menorrhagia	22(16.3%)	6(30.0%)		4(33.3%)		18(18.6%)	0.242
FSH (IU/L)	6.16 (5.26, 7.00)	5.95 (5.49, 7.25)		6.14 (5.72, 6.71)		6.40 (5.27, 7.54)	0.473
LH (IU/L)	11.68 (8.45, 16.48) ^**d**^	9.55 (4.41, 16.10)		7.19 (3.44, 14.33)		10.08 (6.28, 15.37) ^**a**^	0.010
LH/FSH ratio	1.95 (1.41, 2.82) ^**d**^	1.48 (0.77, 2.34)		1.03 (0.65, 2.74)		1.75 (1.03, 2.24) ^**a**^	0.002
TT (ng/mL)	0.71 ± 0.23 ^**d**^	0.60 ± 0.24		0.59 ± 0.24		0.45 ± 0.13 ^**a**^	<0.001
PRL (ng/mL)	12.00 (9.14, 15.15)	11.20 (7.03, 25.23)		12.60 (9.85, 16.53)		12.00 (7.88, 16.13)	0.835
SHBG (nmol/L)	32.90 (21.10, 55.40)	36.75 (24.45, 49.55)		34.20 (20.83, 52.48)		31.60 (20.05, 51.30)	0.946
FAI	6.7(4.0, 14.1) ^**d**^	5.2(2.9, 9.7)		5.3(3.3, 10.9)		4.8(2.8, 8.1) ^**a**^	0.004
AMH (ng/mL)	10.11 (6.58, 13.46) ^**b**^	5.75 (3.98, 10.77) ^**a**^		6.80 (3.95, 12.58)		8.38 (5.52, 12.39)	0.020
FBG (mmol/L)	5.0 (4.7, 5.2)	5.1 (4.6, 5.2)		5.2 (4.8, 5.4)		5.0 (4.8, 5.3)	0.217
2 h-PG (mmol/L)	6.1 (5.2, 7.0)	5.7 (4.8, 6.8)		5.9 (5.5, 6.8)		6.0 (5.4, 7.2)	0.597
FINS(uIU/mL)	9.6 (6.4,15.6)	9.4 (6.6, 17.0)		10.3 (7.8, 26.9)		10.0 (7.4, 16.1)	0.472
2 h-PI (uIU/mL)	43.6 (27.3, 89.7)	45.9 (33.4, 101.0)		38.6 (24.8, 87.8)		39.9 (27.0, 72.8)	0.414
HOMA-IR	2.07 (1.37, 3.52)	1.94 (1.34, 3.89)		2.39 (1.67, 6.27)		2.18 (1.70, 3.80)	0.368
TG (mmol/L)	0.96 (0.60, 1.26)	0.96 (0.48, 1.35)		1.00 (0.81, 1.24)		0.96 (0.67, 1.45)	0.428
TC (mmol/L)	4.74 (4.15, 5.18)	4.58 (3.98, 4.79)		4.94 (4.72, 5.44)		4.74 (4.31, 5.27)	0.212
LDL-C (mmol/L)	2.90 (2.42, 3.25)	2.79 (2.29, 3.23)		3.07 (2.87, 3.59)		2.90 (2.47, 3.39)	0.187
HDL-C (mmol/L)	1.32 (1.14, 1.48)	1.32 (1.18, 1.51)		1.32 (1.10, 1.70)		1.32 (1.11, 1.47)	0.894

Data are presented as median (interquartile range), mean ± standard deviation or N (percentage)

PCOS, polycystic ovary syndrome; BMI, body mass index; WHR, waist to hip ratio; mF-G: modified Ferriman-Gallwey; FSH, follicle stimulating hormone; LH, luteinizing hormone; TT, total testosterone; PRL, prolactin; SHBG, sex hormone-binding globulin; FAI, free androgen index; AMH, anti-Müllarian hormone; FBG, fasting blood glucose; 2h-PG, 2-hour postprandial blood glucose; FINS, fasting insulin; 2h-PI, 2-hour postprandial insulin; HOMA-IR, homeostasis model of assessment-insulin resistance; TG, triacylglycerol; TC, total cholesterol; LDL-C, low-density lipoprotein cholesterol; HDL-C, high-density lipoprotein cholesterol

PCOS-A = OA+HA+PCOM; PCOS-B = OA+HA; PCOS-C = HA+PCOM; PCOS-D = OA+PCOM; OA, oligo-anovulation; HA, hyperandrogenism; PCOM, polycystic ovarian morphology

Kruskal-Wallis test is used to compare the differences between phenotypes, followed by pairwise comparisons as post hoc analysis. a, b, c, d indicate statistically significant differences when compared to phenotype A, B, C, and D, respectively (*P*<0.05)

The Kruskal-Wallis test was employed to compare various indicators across these four PCOS phenotypes, followed by pairwise comparisons. The results (Table 2) revealed no statistically significant differences in age, BMI, WHR, facial acne, chest acne, back acne, infertility percentage, menstrual duration, menorrhagia percentage, FSH levels, PRL levels, SHBG levels, FBG levels, FINS levels, 2 h-PG levels, 2 h-PI levels, HOMA-IR, TG levels, TC levels, LDL-C levels, or HDL-C levels among the four phenotypes (*P* > 0.05).

However, notable differences were observed in the mF-G score, alopecia, ovarian volume, age at menarche, times of menstrual cycles, LH levels, LH/FSH ratio, TT levels, FAI, and AMH levels across the different phenotypes (*P* < 0.05). Specifically, Phenotype D (2[1, 3]) exhibited a significantly lower median mF-G score compared to the other three phenotypes (Phenotype A: 5[2, 8]; Phenotype B: 7[5, 11]; Phenotype C: 6[4, 8]; *P* < 0.05). Phenotype B (6.68 [5.50, 7.99] cm^3^) demonstrated a significantly lower median ovarian volume than Phenotype A (9.43 [7.97, 13.10] cm^3^) and Phenotype D (9.00 [7.21, 11.44] cm^3^, *P* < 0.05). Phenotype C (12 [11, 12]) had a significantly higher median times of menstrual cycles than the other three phenotypes (Phenotype A: 6 [4, 10]; Phenotype B: 8 [4, 10]; Phenotype D: 8 [5, 10]; *P* < 0.05). When compared to Phenotype A, Phenotype D showed significantly lower median levels of TT (0.71 ± 0.23 vs. 0.45 ± 0.13 ng/mL, *P* < 0.05), LH (11.68 [8.45, 16.48] vs. 10.08 [6.28, 15.37] IU/L, *P* < 0.05), LH/FSH ratio (1.95 [1.41, 2.82] vs. 1.75 [1.03, 2.24], *P* < 0.05), and FAI (6.7 [4.0, 14.1] vs. 4.8 [2.8, 8.1], *P* < 0.05). Furthermore, Phenotype B had a significantly lower median AMH level than Phenotype A.

### ROC curve of AMH for predicting PCOS

**Fig. 1:**
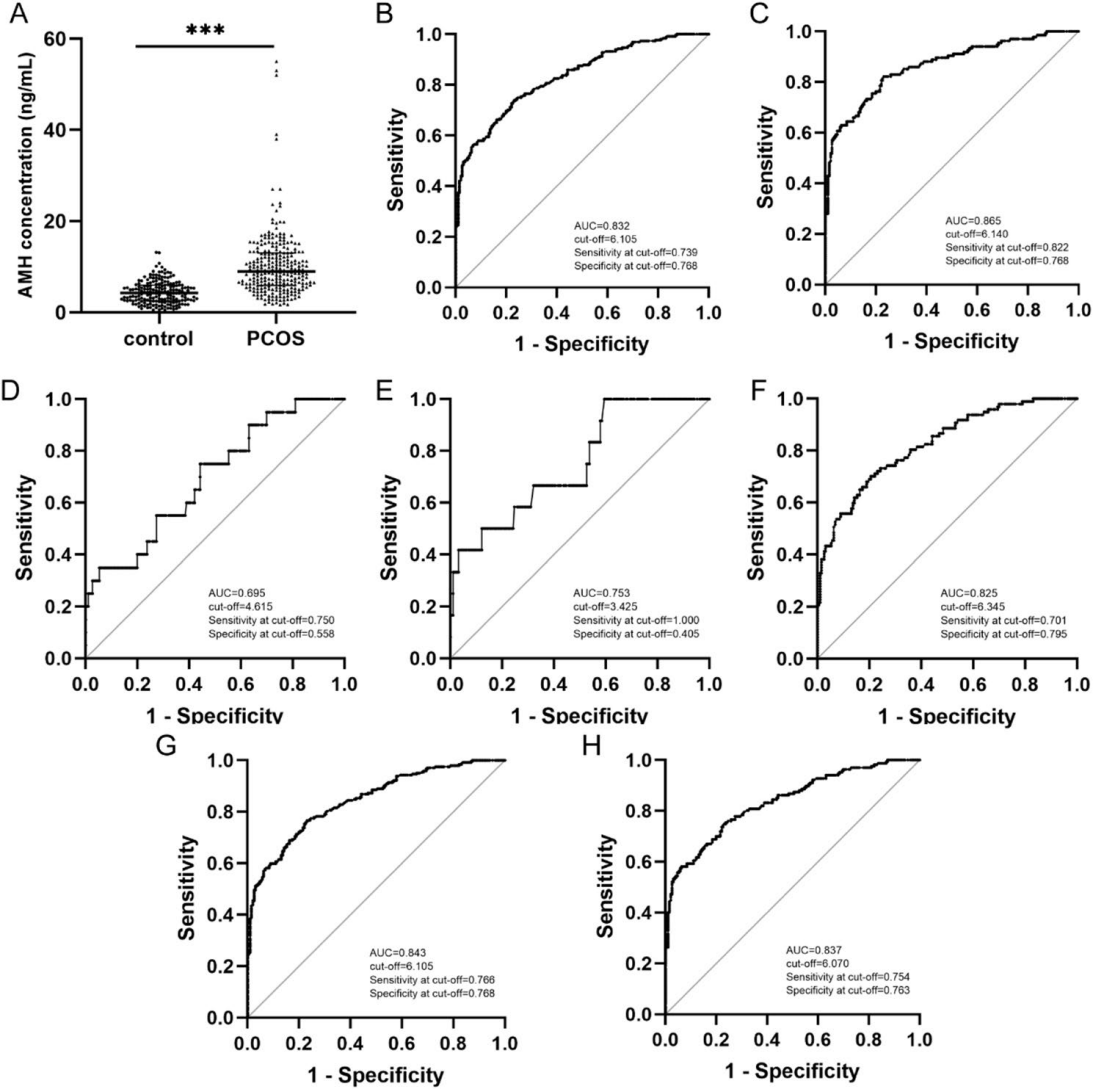
ROC curve of AMH for predicting PCOS. Scatter plot of serum anti-Müllarian hormone (AMH) concentration distribution (**A**) in the control group and PCOS group. Receiver operating characteristic (ROC) curves of serum AMH for predicting PCOS Status (**B**), PCOS phenotype A (**C**), PCOS phenotype B (**D**), PCOS phenotype C (**E**), PCOS phenotype D (**F**), PCOS phenotype A, C, D (**G**), and PCOS phenotype A, B, C (**H**), respectively. The scatter plot represents the median and interquartile range, Mann-Whitney U test is utilized for analysis AMH concentration. ***P<0.001. AUC, area under the curve; PCOS, polycystic ovary syndrome

As depicted in Fig. 1A, serum AMH levels were significantly elevated in the PCOS group, being 2.1 times higher than those in the normal control group (4.29 [2.51, 5.99] vs. 8.97 [5.94, 13.03] ng/mL, *P*<0.001). We plotted ROC curves to assess the diagnostic efficacy of serum AMH for PCOS, various PCOS phenotypes, and PCOM, quantified by calculating the AUC. The results indicated that the cut-off value of AMH for diagnosing PCOS was 6.105 ng/mL, with an AUC (95% CI) of 0.832 (0.796, 0.868), a sensitivity of 0.739, and a specificity of 0.768, demonstrating high accuracy in distinguishing PCOS patients from healthy controls (Fig. 1B).

Given the notable disparities in clinical, endocrine, and metabolic features across various PCOS phenotypes, especially Phenotype A which frequently manifests a more severe presentation (Table 2), we individually plotted ROC curves for serum AMH to diagnose distinct PCOS subtypes and assessed their diagnostic efficacy. For PCOS Phenotype A, the cut-off value of AMH was 6.140 ng/mL, with an AUC (95% CI) of 0.865 (0.824, 0.906) (Fig. 1C). For PCOS Phenotype B, the cut-off value was 4.615 ng/mL, with an AUC (95% CI) of 0.695 (0.575, 0.816) (Fig. 1D). For PCOS Phenotype C, the cut-off value was 3.425 ng/mL, with an AUC (95% CI) of 0.753 (0.613, 0.893) (Fig. 1E). For PCOS Phenotype D, the cut-off value was 6.345 ng/mL, with an AUC (95% CI) of 0.825 (0.774, 0.875) (Fig. 1F). Furthermore, we plotted the ROC curve for AMH in diagnosing PCOM-positive phenotypes (phenotypes A, C, D), with a cut-off value of 6.105 ng/mL and an AUC (95% CI) of 0.843 (0.808, 0.879) (Fig. 1G). Additionally, we also plotted the ROC curve for AMH in diagnosing HA-positive phenotypes (phenotypes A, B, C), which had a cutoff value of 6.070 ng/mL and an AUC (95% CI) of 0.837(0.795,0.878) (Fig. 1H).

### Diagnostic efficacy of AMH combined with other sex hormone indicators for PCOS

We further evaluated the diagnostic potential of combining AMH with other indicators (including LH, TT, PRL, and additional sex hormones) for PCOS identification. The results, as depicted in Table 3, indicate that the AUC for diagnosing PCOS ranged from 0.839 to 0.905 when AMH was combined with individual indicators such as the LH/FSH ratio, LH, FSH, TT, SHBG, and FAI. Notably, when AMH was combined with multiple sex hormone (FSH, LH, PRL, T, SHBG), the AUC (95% CI) increased to 0.923 (0.899, 0.947), with a sensitivity of 0.841 and a specificity of 0.863.

**Table 3:** Diagnostic efficacy of AMH combined with other sex hormone indicators for PCOS

Indicators	AUC (95%CI)	sensitivity	specificity	Youden index	*P*-value
AMH + LH/FSH ratio	0.905(0.878, 0.932)	0.811	0.879	0.690	<0.001
AMH + LH	0.881(0.850, 0.912)	0.777	0.889	0.666	<0.001
AMH + PRL	0.859(0.826, 0.892)	0.731	0.826	0.557	<0.001
AMH + FSH	0.843(0.808, 0.877)	0.621	0.905	0.526	<0.001
AMH + TT	0.839(0.804, 0.874)	0.591	0.926	0.517	<0.001
AMH + SHBG	0.851(0.817, 0.885)	0.705	0.842	0.547	<0.001
AMH + FAI	0.876(0.845, 0.907)	0.742	0.847	0.590	<0.001
AMH + FSH + LH + PRL + T + SHBG	0.923(0.899, 0.947)	0.841	0.863	0.704	<0.001

PCOS, polycystic ovary syndrome; AMH, anti-Müllarian hormone; FSH, follicle stimulating hormone; LH, luteinizing hormone; TT, total testosterone; PRL, prolactin; SHBG, sex hormone-binding globulin; FAI, free androgen index; AUC, area under the curve. Youden index = sensitivity + specificity – 1

### Correlation between AMH and other variables in PCOS group

Further exploration of the correlation between serum AMH levels and other indicators in patients with PCOS was conducted using Spearman’s correlation analysis. The data presented in Table 4 reveal that age (*r* = -0.148, *P* = 0.016), mF-G score (*r* = -0.125, *P* = 0.043), times of cycle (*r* = -0.186, *P* = 0.002), FINS (*r* = -0.180, *P* = 0.003), HOMA-IR (*r* = -0.169, *P* = 0.006), and 2 h-PI (*r* = -0.146, *P* = 0.017) exhibited negative correlations with AMH. Conversely, ovarian volume (*r* = 0.253, *P* < 0.001), FSH (*r* = 0.170, *P* = 0.006), LH (*r* = 0.510, *P* < 0.001), LH/FSH ratio (*r* = 0.448, *P* < 0.001), and TT (*r* = 0.182, *P* = 0.003) showed positive correlations. However, no significant correlations were observed between serum AMH and BMI, WHR, PRL, SHBG, FAI, FBG, 2 h-PG, TG, TC, LDL-C, or HDL-C (*P* > 0.05).

**Table 4:** Correlation between AMH and other variables in PCOS group

Variables	AMH	
	*r*	*P*
Age	-0.148	0.016
BMI	-0.120	0.052
WHR	-0.026	0.669
mF-G score	-0.125	0.043
Facial acne	-0.070	0.258
Chest acne	-0.036	0.560
Back acne	-0.053	0.393
Alopecia	-0.035	0.571
ovarian volume	0.253	<0.001
Times of Cycle	-0.186	0.002
FSH	0.170	0.006
LH	0.510	<0.001
LH/FSH ratio	0.448	<0.001
PRL	-0.111	0.073
TT	0.182	0.003
SHBG	0.099	0.109
FAI	-0.017	0.781
FBG	-0.003	0.961
2 h-PG	-0.095	0.122
FINS	-0.180	0.003
2 h-PI	-0.146	0.017
HOMA-IR	-0.169	0.006
TG	-0.031	0.621
TC	0.067	0.280
LDL-C	0.029	0.640
HDL-C	0.045	0.468

PCOS, polycystic ovary syndrome; BMI, body mass index; WHR, waist to hip ratio; mF-G: modified Ferriman-Gallwey; FSH, follicle stimulating hormone; LH, luteinizing hormone; TT, total testosterone; PRL, prolactin; SHBG, sex hormone-binding globulin; FAI, free androgen index; AMH, anti-Müllarian hormone; FBG, fasting blood glucose; 2h-PG, 2-hour postprandial blood glucose; FINS, fasting insulin; 2h-PI, 2-hour postprandial insulin; HOMA-IR, homeostasis model of assessment-insulin resistance; TG, triacylglycerol; TC, total cholesterol; LDL-C, low-density lipoprotein cholesterol; HDL-C, high-density lipoprotein cholesterol

Using Spearman’s correlation analysis (*P*<0.05)

Our analysis revealed a significant age difference between BMI subgroups (26 vs. 29 years, *P* = 0.001). To account for potential age confounding, we performed age-stratified analyses. As shown in Supplementary Table 2, the relationship between AMH and BMI varied across age groups. While the overall cohort showed a borderline inverse correlation (*r*=-0.120, *P* = 0.052), this association reached significance in women < 35 years (*r*=-0.127, *P* = 0.046). The ≥ 35 years subgroup demonstrated a stronger negative trend (*r*=-0.242) that was not statistically significant, likely due to limited sample size (*n* = 15).

### AMH levels in control and PCOS groups across different age subgroups

**Fig. 2:**
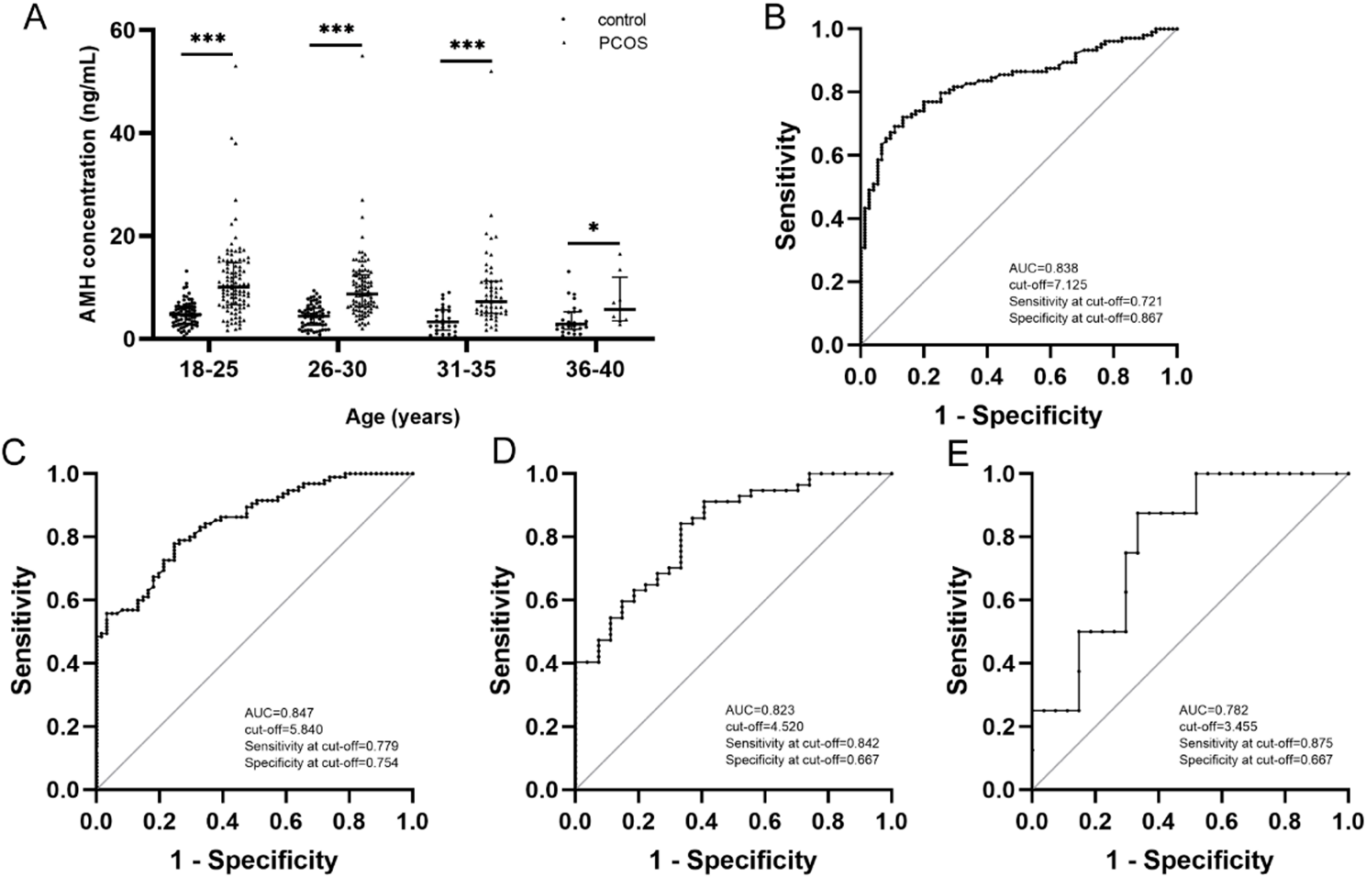
ROC Curve of AMH for predicting PCOS status in the different age groups. Scatter plot of serum anti-Müllarian hormone (AMH) concentration distribution (**A**) in the control group and PCOS group across different age subgroups. Receiver operating characteristic (ROC) curves of serum AMH for predicting polycystic ovary syndrome (PCOS) status in the age groups 18–25 years (**B**), 26–30 years (**C**), 31–35 years (**D**), 36–40 years (**E**), respectively. The scatter plot represents the median and interquartile range, Mann-Whitney U test is utilized for analysis. *P<0.05, ***P<0.001. AUC, area under the curve

Building upon well-established evidence of age-related decline in AMH levels and employing validated methodological approaches from prior AMH studies, we categorized participants into four age subgroups (18–25, 26–30, 31–35, and 36–40 years) to evaluate diagnostic performance [12, 19, 24–26]. Our findings further confirmed an inverse correlation between AMH levels and age in PCOS patients (*r*=-0.148, *P* = 0.016; Table 4). Within each age subgroup (Fig. 2A; Table 5), the median serum AMH levels were significantly elevated in the PCOS group compared to the control group (18–25 years: 4.74 [3.01, 6.32] vs. 10.10 [6.64, 14.85] ng/mL, *P* < 0.001; 26–30 years: 4.43 [2.67, 6.02] vs. 8.72 [6.23, 12.44] ng/mL, *P* < 0.001; 31–35 years: 3.34 [1.68, 5.64] vs. 7.21 [4.99, 11.24] ng/mL, *P* < 0.001; 36–40 years: 2.87 [1.89, 5.29] vs. 5.71 [3.54, 11.98] ng/mL, *P* = 0.017). Both the control and PCOS groups exhibited a gradual decrease in median serum AMH levels with advancing age. While significant differences were observed in the overall age-stratified analysis, pairwise comparisons between adjacent age groups did not remain statistically significant after multiple testing correction in both cohorts.

**Table 5:** AMH levels in control and PCOS groups across different age subgroups

Age(years)	Control		PCOS		*P*-value
	*N*	Median (IQR) (ng/mL)	*N*	Median (IQR) (ng/mL)	
18–25	75	4.74 (3.01, 6.32)	104	10.10 (6.64, 14.85)	<0.001
26–30	61	4.43(2.67, 6.02)	95	8.72(6.23, 12.44)	<0.001
31–35	27	3.34(1.68, 5.64)	57	7.21(4.99, 11.24)	<0.001
36–40	27	2.87 (1.89, 5.29)	8	5.71 (3.54, 11.98)	0.017

Data are presented as median (interquartile range)

Mann-Whitney U test is utilized for analysis (*P*<0.05)

ROC curves were plotted to assess the diagnostic efficacy of AMH in identifying PCOS across different age subgroups. Notably, the cut-off values for AMH in diagnosing PCOS decreased with increasing age (Fig. 2B-E). Specifically, in the 18–25 years subgroup, the ROC-AUC (95% CI) for AMH in diagnosing PCOS was 0.838 (0.780, 0.896), with a cut-off value of 7.125 ng/mL (Fig. 2B). In the 26–30 years subgroup, the ROC-AUC (95% CI) was 0.847 (0.789, 0.906), with a cut-off value of 5.840 ng/mL (Fig. 2C). For the 31–35 years subgroup, the ROC-AUC (95% CI) was 0.823 (0.731, 0.915), with a cut-off value of 4.520 ng/mL (Fig. 2D). Lastly, in the 36–40 years subgroup, the ROC-AUC (95% CI) was 0.782 (0.623, 0.942), with a cut-off value of 3.455 ng/mL (Fig. 2E).

The diagnostic efficacy of AMH in identifying PCOS varied across the different age subgroups. Specifically, the median ROC-AUC in the 18–25 years and 26–30 years subgroups surpassed that of the overall population. Conversely, the median ROC-AUC in the 31–35 years and 36–40 years subgroups was inferior to that of the overall population.

### AMH levels in control and PCOS groups across different BMI subgroups

**Fig. 3:**
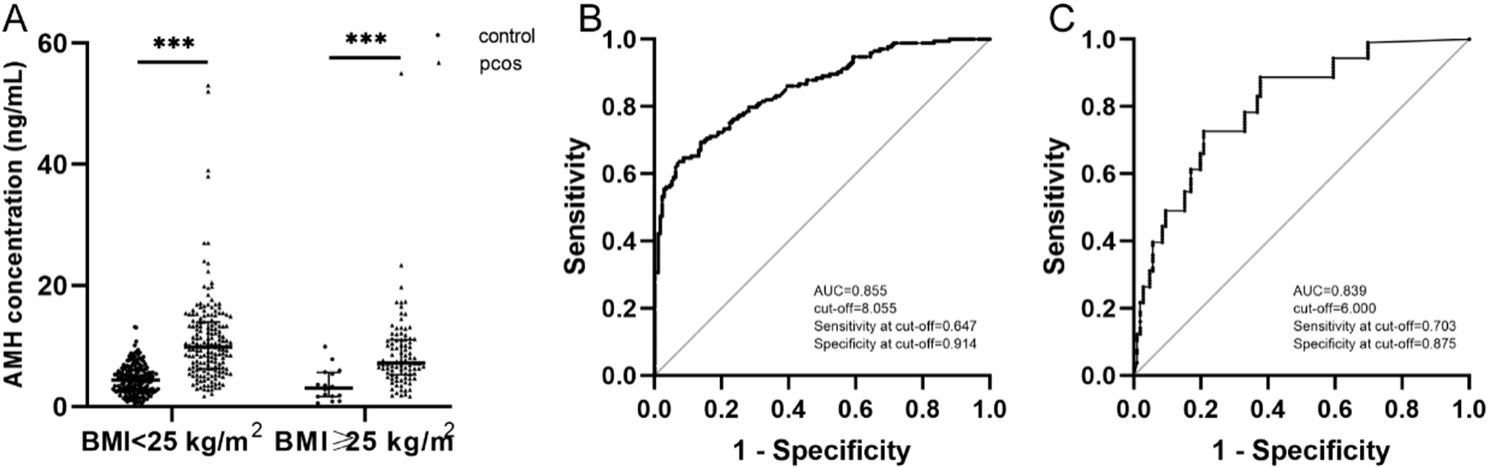
ROC curve of AMH for predicting PCOS status in the different BMI groups. Scatter plot of serum anti-Müllarian hormone (AMH) concentration distribution (**A**) in the control group and PCOS group across different BMI subgroups. Receiver operating characteristic (ROC) curves of serum AMH for predicting polycystic ovary syndrome (PCOS) status in the BMI groups BMI<25 kg/m^2^ (**B**), BMI ≥ 25 kg/m^2^ (**C**), respectively. The scatter plot represents the median and interquartile range, Mann-Whitney U test is utilized for analysis. ***P<0.001. AUC, area under the curve

BMI is a key factor influencing AMH levels in PCOS patients [20]. The underweight/normal weight group consisted of 174 controls and 173 patients with PCOS, with the median serum AMH level being significantly higher in the PCOS group compared to the control group (4.37 [2.63, 6.07] vs. 9.88 [6.28, 13.98] ng/mL, *P* < 0.001). Similarly, the overweight/obese group comprised 16 controls and 91 patients with PCOS, exhibiting a significantly elevated median serum AMH level in the PCOS group (3.10 [1.70, 5.63] vs. 7.18 [5.29, 11.09] ng/mL, *P* < 0.001) (Table 6; Fig. 3A). Additionally, within the PCOS population, the median serum AMH level was significantly lower in the overweight/obese group compared to the underweight/normal weight group (9.88 [6.28, 13.98] vs. 7.18 [5.29, 11.09] ng/mL, *P* < 0.05) (Table 6).

**Table 6:** AMH levels in control and PCOS groups across different BMI subgroups

	Control		PCOS		*P*-value
	*N*	Median (IQR)	*N*	Median (IQR)	
BMI < 25 kg/m^2^	174	4.37(2.63, 6.07)	173	9.88(6.28, 13.98)	<0.001
BMI ≥ 25 kg/m^2^	16	3.10(1.70, 5.63)	91	7.18(5.29, 11.09) ^a^	<0.001

Data are presented as median (interquartile range)

Mann-Whitney U test is utilized for analysis (*P*<0.05). Within both the PCOS group and the control group, comparisons of AMH levels were conducted between different BMI subgroups. a revealed statistically significant difference in AMH levels between the BMI ≥ 25 kg/m^2^ subgroup and the BMI < 25 kg/m^2^ subgroup(*P*=0.002)

ROC curves for PCOS diagnosis using AMH were constructed separately for the underweight/normal weight and overweight/obese groups. In the underweight/normal weight group, the ROC-AUC for PCOS diagnosis with AMH was 0.855, with a cut-off value of 8.055 ng/mL (Fig. 3B). In contrast, the overweight/obese group exhibited a ROC-AUC of 0.839 for PCOS diagnosis with AMH, with a cut-off value of 6.000 ng/mL (Fig. 3C). The ROC-AUC in the underweight/normal weight group exceeded that of the overall population, whereas it was lower in the overweight/obese group.

The observed reduction in AMH levels among overweight/obese PCOS patients may reflect metabolic influences on ovarian function, as excess adiposity has been associated with altered granulosa cell activity and follicular development [33, 34].

### AMH as an independent risk factor for infertility in patients with PCOS

Building upon the observed variations in AMH levels across age and BMI subgroups, we further investigated whether these AMH differences might have clinical implications for fertility outcomes in PCOS patients. Patients with PCOS were divided into two subgroups based on the presence of infertility: the PCOS without infertility group included 206 subjects, while the PCOS with infertility group comprised 58 subjects. A comparison of baseline characteristics between these two subgroups is presented in Supplementary Table 1. Compared to the PCOS without infertility group, the PCOS with infertility group exhibited significantly higher median values for age (26 [23, 30] vs. 30 [28, 33] years, *P*<0.001), BMI (22.6 [20.5, 25.8] vs. 25.2 [22.4, 27.7] kg/m^2^, *P* = 0.001), WHR (0.81 [0.77, 0.84] vs. 0.83 [0.79, 0.87], *P* = 0.004), FAI (5.8 [3.2, 9.6] vs. 7.8 [4.2, 13.6], *P* = 0.016), FBG (5.0 [4.7, 5.2] vs. 5.1 [4.9, 5.3] mmol/L, *P* = 0.002), FINS (9.6 [6.7, 14.6] vs. 13.6 [6.9, 19.6] µIU/mL, *P* = 0.044), HOMA-IR (2.07 [1.47, 3.26] vs. 3.14 [1.49, 5.02], *P* = 0.024), and TG (0.96 [0.61, 1.21] vs. 1.20 [0.91, 1.88] mmol/L, *P* = 0.001). Conversely, significantly lower median values were observed for SHBG levels, and HDL-C (*P* < 0.05).

Median serum AMH levels were 8.70 ng/mL and 9.76 ng/mL in the PCOS without infertility and PCOS with infertility groups, respectively, showing no statistically significant difference between the two groups (*P* = 0.409).

Multivariate logistic regression analysis identified three independent predictors of infertility in PCOS patients (Table 7): increasing age (OR = 1.242, 95% CI: 1.136–1.359, *P* < 0.001), elevated AMH levels (OR = 1.058, 95% CI: 1.010–1.109, *P* = 0.018), and decreased HDL-C levels (OR = 0.098, 95% CI: 0.019–0.493, *P* = 0.005).

**Table 7:** Odds ratios for infertility prediction in PCOS group after multivariate logistic regression

Variables	OR	95%CI lower bound	95%CI upper bound	*P*-value
Age	1.242	1.136	1.359	<0.001*
BMI	1.020	0.915	1.137	0.721
WHR	3.280	0.002	6307.053	0.758
mF-G score	0.929	0.832	1.038	0.193
SHBG	0.997	0.982	1.013	0.734
FAI	1.010	0.949	1.075	0.748
AMH	1.058	1.010	1.109	0.018*
FBG	1.798	0.856	3.775	0.121
FINS	0.952	0.906	1.000	0.052
TG	1.525	0.800	2.907	0.200
HDL-C	0.098	0.019	0.493	0.005*

BMI, body mass index; WHR, waist to hip ratio; mF-G: modified Ferriman-Gallwey; SHBG, sex hormone-binding globulin; FAI, free androgen index; AMH, anti-Müllarian hormone; FBG, fasting blood glucose; FINS, fasting insulin; TG, triacylglycerol; HDL-C, high-density lipoprotein cholesterol

## Discussion

Our study investigated the diagnostic value of serum AMH for PCOS in Chinese women of reproductive age, with analyses stratified by age and BMI. We observed significantly higher AMH levels in PCOS patients compared to controls (8.97 vs. 4.29 ng/mL; *P* < 0.001), with 6.105 ng/mL showing optimal diagnostic accuracy (AUC = 0.832). The diagnostic performance varied across PCOS phenotypes, being most robust for Phenotype A (AUC = 0.865). Combining AMH with other hormonal markers further enhanced diagnostic efficacy (AUC = 0.923). Notably, diagnostic accuracy was better in younger women and those with lower BMI. AMH levels showed positive correlations with ovarian volume and LH levels, while demonstrating negative associations with age and insulin resistance markers. Importantly, AMH emerged as an independent risk factor for infertility (OR = 1.058; *P* = 0.018). These findings not only support existing evidence but also provide clinically relevant, population-specific diagnostic thresholds and new insights into how metabolic factors influence AMH’s diagnostic performance in PCOS.

This study compared differences in clinical, endocrine, and metabolic indicators between the PCOS and control groups, as well as among the four PCOS phenotypes. The PCOS group exhibited a higher proportion of overweight / obese individuals, accompanied by notable abnormalities in clinical, endocrine, and metabolic characteristics.

In our cohort, Phenotype A (51.1%) predominated over Phenotype D (36.7%), contrasting with some Chinese studies reporting reversed prevalence. These variations highlight PCOS heterogeneity across populations [35, 36]. Consistent with prior research, our data revealed that among the four phenotypes, Phenotype A exhibited more severe features, including higher ovarian volume, LH levels, LH/ FSH ratio, TT levels, FAI, and AMH levels, suggesting potentially more severe ovarian dysfunction in patients with Phenotype A [31, 37, 38]. However, no differences were observed in BMI, FBG levels, FINS levels, 2 h-PG levels, 2 h-PI levels, HOMA-IR, TG levels, TC levels, LDL-C levels, or HDL-C levels among the four phenotypes. These results indicate that there are significant differences in some clinical androgenic symptoms and endocrine characteristics among the four PCOS phenotypes, while there are no differences in metabolic characteristics except for FAI. This suggests that AMH may help distinguish different subtypes of PCOS, providing a basis for the formulation of personalized treatment plans. Consistent with previous studies, our data showed that AMH levels were significantly higher in patients with PCOS compared to healthy controls, being 2.1 times higher in the PCOS group [13–15, 38]. AMH is primarily secreted by granulosa cells of preantral and small antral follicles, and its levels directly reflect the number and developmental status of small follicles within the ovaries [11]. Elevated AMH levels in PCOS patients likely result from multiple interacting mechanisms. Hyperandrogenism promotes small antral follicle growth while impairing maturation, leading to follicular accumulation and increased AMH production [16, 39], consistent with our observed AMH-testosterone correlation (*r* = 0.182, *P* = 0.003). Granulosa cells in PCOS demonstrate enhanced AMH secretion, potentially through both intrinsic changes and LH stimulation, as supported by our strong LH-AMH association (*r* = 0.510, *P* < 0.001) and prior studies [16, 40]. AMH may further sustain this abnormal follicular milieu by suppressing aromatase activity and FSH sensitivity [12, 41].

The established AMH cutoff of 6.105 ng/mL demonstrated robust diagnostic performance for PCOS in Chinese reproductive-aged women (sensitivity 0.739, specificity 0.768, AUC 0.832), consistent with previous research [18, 38, 42, 43]. This threshold provides clinicians with a reliable biochemical marker that complements existing diagnostic criteria, particularly valuable when ultrasound findings are inconclusive or unavailable.

AMH showed differential diagnostic accuracy across PCOS phenotypes. It performed best for Phenotype A (AUC = 0.865) and PCOM-positive phenotypes (AUC = 0.843), but had limited utility for PCOM-negative cases (AUC = 0.695). While our results for hyperandrogenic phenotypes (AUC = 0.837) aligned with most studies, they contrasted with Li et al.‘s report (AUC = 0.66) [44], possibly reflecting population differences in phenotypic expression. These findings indicate that AMH can not only identify PCOS but also differentiate between various PCOS phenotypes, particularly in identifying the more severe Phenotype A. The observed variations in AMH diagnostic performance across PCOS phenotypes carry important clinical implications. Phenotype A (classic PCOS) typically presents with the most severe metabolic and reproductive complications, while Phenotype D (ovulatory PCOS) often exhibits milder manifestations. These phenotypic differences directly inform prognosis and therapeutic decision-making, as patients with hyperandrogenism (Phenotypes A-C) may require more aggressive androgen-lowering interventions, whereas those with oligo-anovulation alone (Phenotype D) might benefit primarily from ovulation induction. Our finding that AMH demonstrates particularly strong diagnostic accuracy for Phenotype A (AUC 0.865) suggests its potential utility in identifying patients who may need comprehensive metabolic and reproductive management.

The diagnostic challenges in Phenotype B underscore the continued importance of a comprehensive approach emphasizing clinical and biochemical markers of hyperandrogenism and oligo-anovulation. The lower AUC observed in Phenotype B likely reflects the distinct pathogenesis of this subgroup, in which non-follicular mechanisms including hypothalamic-pituitary dysfunction and metabolic disturbances appear to predominate [3]. While AMH measurement enhances diagnostic accuracy in PCOM-positive cases, its limited performance in Phenotype B necessitates cautious interpretation and reinforces the ongoing value of Rotterdam criteria for this phenotype. These findings emphasize the importance of developing phenotype-specific diagnostic strategies, especially for PCOM-negative presentations. Further research should investigate whether combining AMH with other relevant markers, such as androgen level indices, could improve diagnostic precision for Phenotype B, potentially enabling more tailored diagnostic approaches across the PCOS spectrum.

Additionally, when serum AMH levels were combined with other sex hormone indicators (such as LH, FSH, testosterone, etc.), the diagnostic efficacy further improved, with the highest AUC reaching 0.923. These findings suggest that combining serum AMH levels with one or more sex hormone indicators enhances the diagnostic efficacy of PCOS compared to relying solely on AMH levels (AUC = 0.832). This provides valuable medical insights for PCOS diagnosis using serum AMH combined with sex hormones and holds promise for clinical application. This suggests that combined detection can reduce missed and misdiagnosed cases, enhancing diagnostic accuracy. Other studies have also affirmed the diagnostic value of AMH combined with other indicators for PCOS [45]. For patients where transvaginal ultrasound is not preferred or feasible, and when transabdominal ultrasound proves suboptimal (particularly in obese individuals), AMH measurement provides a valuable diagnostic alternative [12, 46].

Our data revealed that AMH levels in the PCOS group were positively correlated with ovarian volume, LH levels, LH/FSH ratio, and TT levels, while negatively correlated with age and menstrual cycle frequency. These findings are consistent with other research results [15, 31, 45], suggesting that AMH may regulate reproductive function by influencing follicular development and hormone secretion, providing new insights into the pathophysiological mechanisms of PCOS. The relationship between AMH and insulin resistance markers showed more complexity. While we observed negative correlations with FINS, 2 h-PI, and HOMA-IR, the literature remains divided. Some studies report positive correlations [47, 48], while others found no association [49, 50]. These discrepancies may be attributed to several factors including population characteristics such as BMI distribution and ethnic background, methodological differences in assessing insulin resistance, and variations in PCOS phenotype proportions across study cohorts.

Through subgroup analyses stratified by age and BMI, we evaluated the clinical applicability of serum AMH for PCOS diagnosis. The diagnostic performance of AMH was strongest in younger women (18–30 years) and declined with advancing age, suggesting that age-specific reference ranges may improve diagnostic accuracy. Both our findings and those of other studies emphasize the importance of considering age when establishing diagnostic criteria for PCOS [17, 42]. Similarly, while AMH maintained diagnostic utility across BMI categories, its levels and discriminatory power were consistently higher in underweight/normal weight women compared to overweight/obese individuals, consistent with previous research [33].

The relationship between obesity and AMH levels in PCOS patients has been documented in multiple clinical studies, though population-specific variations exist. Nouri, Mohammad et al. demonstrated that overweight PCOS patients exhibit both lower serum AMH levels and reduced AMHR-II expression in granulosa cells compared to normal-weight patients, suggesting obesity may directly impair AMH production [34]. However, this association appears population-specific, as Moy, Vicky et al. found BMI negatively correlated with AMH only in Caucasian women, not in African American, Hispanic, or Asian populations [51]. Furthermore, Zeng et al. observed that central adiposity (waist circumference) showed stronger inverse correlations with AMH than BMI-based obesity measures [22]. These findings collectively suggest that the obesity-AMH relationship in PCOS may be mediated through both direct ovarian effects and metabolic pathways that vary across ethnic groups. Further investigation is needed to elucidate these complex interactions.

The lack of a significant correlation between BMI and AMH in the Spearman analysis (*r*=-0.120, *P* = 0.052), while observing significant differences between BMI subgroups (*P* = 0.002), may initially appear contradictory. However, the absence of a linear correlation does not preclude the existence of threshold effects or non-linear relationships between BMI and AMH levels. Our subgroup analysis revealed that although AMH remained elevated in PCOS patients across BMI categories, overweight/obese women showed significantly lower AMH levels than their normal-weight counterparts (7.18 vs. 9.88 ng/mL). This finding suggests that obesity may influence granulosa cell function and AMH production through metabolic pathways rather than simple linear associations [52, 53].

While initial comparisons revealed no significant difference in AMH levels between the non-infertility and infertility groups (8.70 vs. 9.76 ng/mL, *P* = 0.409), subsequent multivariate analysis identified AMH as an independent predictor (OR = 1.058, *P* = 0.018). This discrepancy likely reflects the confounding effects of age and metabolic parameters that were accounted for in the regression model. Specifically, infertile patients were older (30 vs. 26 years) and exhibited worse metabolic profiles, factors which may have masked AMH’s independent contribution in unadjusted analyses. These results underscore that AMH’s predictive value for infertility in PCOS emerges most clearly when considered in the context of other clinical parameters. Serum AMH levels are commonly used to evaluate ovarian reserve and ovarian response for ovarian hyperstimulation of assisted reproductive technology [54, 55]. Elevated AMH levels typically indicate abundant ovarian reserve and an excessive response to ovarian hyperstimulation. Conversely, in PCOS patients, the elevation of AMH due to androgen-induced excessive follicles does not promote ovulation but inhibits aromatase activity and reduces follicle sensitivity to FSH, leading to arrest of follicle growth and development [39–41]. This prevents follicles from maturing and releasing eggs, resulting in ovulation disorders [39]. Mumford, Sunni L et al. found that elevated serum AMH concentrations in women with PCOS correlate with diminished ovarian response to ovulation induction [56]. This finding not only deepens the understanding of the infertility mechanism in PCOS but also provides new insights for fertility management in clinical practice. The well-established association between elevated AMH levels and increased ovarian hyperstimulation syndrome risk in PCOS patients undergoing ovarian stimulation necessitates a careful balance in clinical decision-making [57]. Although more intensive fertility interventions might theoretically enhance conception outcomes in this population, clinicians must judiciously weigh the potential benefits against the significant risks of complications.

While there is a certain research foundation for the application of AMH in the diagnosis of PCOS, this study may provide a new perspective or discovery through stratified assessment by age and BMI, enhancing the innovation of the research. The study focuses on a specific population of Chinese women of reproductive age and attempts to establish AMH diagnostic criteria applicable to this population. This innovative research targeting a specific population contributes to the localization and personalization of PCOS diagnosis, improving the accuracy and applicability of diagnosis.

Despite the rigorous design of this study, there are still some limitations. Firstly, as a single-center case-control study, although strict inclusion and exclusion criteria were followed, the sample may still exhibit certain selection bias and may have specific geographical and population characteristics, which may limit the generalizability of the findings. Second, our cutoff selection prioritized balanced sensitivity and specificity via the Youden index. While this approach is statistically robust, it may differ from thresholds optimized for maximal specificity. Third, our study relied on qualitative ultrasound descriptors rather than precise FNPO counts, as the latter was not routinely documented in clinical practice. This precluded direct correlation analysis between FNPO and AMH levels, particularly across PCOS phenotypes. Although this study used a high-precision Beckman DxI800 automated immunoassay analyzer to measure serum AMH levels, differences in detection methods, equipment, and calibration standards among different laboratories may exist, which could affect the comparison and interpretation of the results. Although the study controlled for confounding factors such as age and BMI, there may still be other unconsidered confounding factors, such as genetic and environmental factors, which could influence the relationship between AMH levels and PCOS, leading to bias in the results. Currently, there are multiple versions of diagnostic criteria for PCOS, with certain differences between them. This study used the Rotterdam diagnostic criteria for classification, but other criteria such as the NIH criteria or Chinese diagnostic guidelines may also be applicable to specific populations. Lastly, this study did not conduct long-term follow-up of PCOS patients and could not assess the relationship between changes in AMH levels and the prognosis of PCOS patients.

The presence of infertility in Phenotype C patients requires careful interpretation, given this phenotype’s exclusion of oligo-/anovulation by diagnostic criteria. This finding suggests these cases likely result from non-ovulatory causes such as male factor infertility or tubal pathology. While including mixed infertility etiologies enhances the clinical relevance of our study, we acknowledge this may have obscured the specific relationship between AMH and PCOS-related ovulatory dysfunction. Future studies with larger sample sizes should stratify analyses by infertility subtype to better characterize AMH’s predictive value for pure PCOS-associated anovulation.

Our findings contribute meaningful clinical insights regarding AMH’s diagnostic value for PCOS in Chinese women. The demonstrated improvement in diagnostic accuracy when combining AMH with other hormonal markers suggests this integrated approach could enhance diagnostic precision in clinical settings. The establishment of phenotype-specific AMH thresholds, particularly for phenotype A, provides clinicians with valuable tools for more accurate PCOS classification. Additionally, our data on the influence of age and BMI on AMH levels offer important practical considerations for result interpretation across different patient subgroups.

Several key areas warrant further investigation. First, multi-center validation studies with larger cohorts are needed to confirm these findings across diverse demographic groups and clinical settings. Second, standardization of AMH assays remains essential to minimize inter-laboratory variation and improve clinical applicability. Third, longitudinal assessments of AMH fluctuations during menstrual cycles and therapeutic interventions could clarify its potential role in monitoring disease course and treatment efficacy. Additionally, while our study employed the Youden index to determine the optimal AMH cutoff, future research should evaluate thresholds prioritizing high specificity to further validate AMH as a surrogate for PCOM. This would complement the Rotterdam criteria’s emphasis on specificity and clarify AMH’s role in settings where minimizing false positives is paramount [3]. Future studies should prioritize standardized FNPO quantification to validate the relationship between AMH and follicular excess in PCOM. Finally, exploration of AMH in combination with emerging biomarkers may further refine PCOS diagnosis and phenotypic characterization.

## Conclusions

This prospective case-control study design delved into the cut-off value of AMH in the diagnosis of PCOS among Chinese women of reproductive age, considering the influence of age and BMI on the diagnostic value of AMH. The combination of AMH with other hormonal indicators further enhanced diagnostic accuracy. The study found that AMH was also significantly associated with various clinical and endocrine indicators, providing valuable insights into PCOS phenotypes and infertility risks. These findings contribute to refining the diagnostic and treatment strategies for polycystic ovary syndrome in the Chinese population.

## Supplementary Information

Below is the link to the electronic supplementary material.

Supplementary Material 1

## Data Availability

The datasets used during the current study are available from the corresponding author on reasonable request.

## Abbreviations

PCOS: Polycystic ovary syndrome
BMI: Body mass index
WHR: Waist to hip ratio
mF-G: Modified Ferriman-Gallwey
FSH: Follicle stimulating hormone
LH: Luteinizing hormone
TT: Total testosterone
PRL: Prolactin
SHBG: Sex hormone-binding globulin
FAI: Free androgen index
AMH: Anti-Müllarian hormone
FBG: Fasting blood glucose
2h-PG: 2-hour postprandial blood glucose
FINS: Fasting insulin
2h-PI: 2-hour postprandial insulin
HOMA-IR: Homeostasis model of assessment-insulin resistance
TG: Triacylglycerol
TC: Total cholesterol
LDL-C: Low-density lipoprotein cholesterol
HDL-C: High-density lipoprotein cholesterol
