# Supplementary material for: Age- and BMI-stratified assessment of serum anti-Müllerian hormone as a biomarker for polycystic ovary syndrome diagnosis in Chinese women
Source: BMC Endocr Disord. 2026 Jan 16;26:46. doi: 10.1186/s12902-025-02136-3 (PMC12895616; doi:10.1186/s12902-025-02136-3)
Supplement: Supplementary file 1 — Supplementary Material 1 [file 12902_2025_2136_MOESM1_ESM.docx]

Supplementary Table 1 Baseline characteristics of PCOS patients with and without Infertility

| Characteristics | PCOS without Infertility(n=206) | PCOS with Infertility (n=58) | *P*-value |
| --- | --- | --- | --- |
|  | **median (IQR) OR N(Percentage)** | **median (IQR) OR N(Percentage)** |  |
| Age(years) | 26 (23, 30) | 30 (28, 33) | ＜0.001 |
| BMI (kg/m^2^) | 22.6(20.5, 25.8) | 25.2(22.4, 27.7) | 0.001 |
| WHR | 0.81 (0.77, 0.84) | 0.83(0.79, 0.87) | 0.004 |
| mFG score | 4(2, 7) | 2(1, 5) | 0.036 |
| Ovarian volume (cm3) | 9.00(7.42, 11.58) | 9.46(7.64, 12.65) | 0.175 |
| Age at Menarche (years) | 13(12, 14) | 13(12, 14) | 0.263 |
| Times of menstrual Cycle | 7(5, 10) | 7(4, 10) | 0.602 |
| Menstrual duration (day) | 6(5,7) | 6(5,7) | 0.742 |
| Menorrhagia | 41(19.9%) | 9(15.5%) | 0.451 |
| FSH (IU/L) | 6.23 (5.26, 7.18) | 6.27 (5.36, 7.36) | 0.649 |
| LH (IU/L) | 10.91 (6.63, 16.04) | 10.75 (7.58, 15.83) | 0.831 |
| LH/FSH ratio | 1.75 (1.15, 2.58) | 1.84 (1.12, 2.47) | 0.805 |
| TT (ng/mL) | 0.58 (0.43, 0.72) | 0.58 (0.47, 0.76) | 0.326 |
| PRL (ng/mL) | 12.00 (8.70, 16.30) | 12.00 (8.74, 14.13) | 0.580 |
| SHBG (nmol/L) | 33.90 (21.88, 54.95) | 25.70 (17.48, 45.43) | 0.025 |
| FAI | 5.8(3.2, 9.6) | 7.8(4.2, 13.6) | 0.016 |
| AMH (ng/mL) | 8.70 (5.75, 12.97) | 9.76 (6.17, 13.67) | 0.409 |
| FBG (mmol/L) | 5.0 (4.7, 5.2) | 5.1 (4.9, 5.3) | 0.002 |
| 2h-PG (mmol/L) | 6.0 (5.3, 6.9) | 6.2 (5.3, 7.4) | 0.252 |
| FINS (uIU/mL) | 9.6 (6.7, 14.6) | 13.6 (6.9, 19.6) | 0.044 |
| 2h-PI (uIU/mL) | 42.4 (27.4, 77.4) | 53.2 (28.4, 104.7) | 0.194 |
| HOMA-IR | 2.07 (1.47, 3.26) | 3.14 (1.49, 5.02) | 0.024 |
| TG (mmol/L) | 0.96 (0.61, 1.21) | 1.20 (0.91, 1.88) | 0.001 |
| TC (mmol/L) | 4.74 (4.22, 5.19) | 4.82 (4.27, 5.14) | 0.507 |
| LDL-C (mmol/L) | 2.90 (2.46, 3.27) | 3.01 (2.36, 3.53) | 0.287 |
| HDL-C (mmol/L) | 1.32 (1.19, 1.54) | 1.18 (1.00, 1.32) | ＜0.001 |

Data are presented as median (interquartile range) OR N (Percentage).

PCOS, polycystic ovary syndrome; BMI, body mass index; WHR**,** waist to hip ratio; mFG: modified Ferriman-Gallwey; FSH, follicle stimulating hormone; LH, luteinizing hormone; TT, total testosterone; PRL, prolactin; SHBG, sex hormone-binding globulin; FAI, free androgen index; AMH, anti-M**ü**llarian hormone; FBG, fasting blood glucose; 2h-PG, 2-hour postprandial blood glucose; FINS, fasting insulin; 2h-PI, 2-hour postprandial insulin; HOMA-IR, homeostasis model of assessment-insulin resistance; TG, triacylglycerol; TC, total cholesterol; LDL-C, low-density lipoprotein cholesterol; HDL-C, high-density lipoprotein cholesterol.

Mann-Whitney U test is utilized for analysis the continuous variables. Categorical variables are analyzed using the chi-square test (*P*＜0.05).

Supplementary Table 2 Correlation between AMH and BMI in different age subgroups of women with PCOS

| Variables | AMH  All PCOS cases | | AMH  ＜35 years group(n=249) | | AMH  ≥35 years group(n=15) | |
| --- | --- | --- | --- | --- | --- | --- |
|  | **r** | ***P*** | **r** | ***P*** | **r** | ***P*** |
| **BMI** | -0.120 | 0.052 | -0.127 | **0.046** | -0.242 | 0.384 |

PCOS, polycystic ovary syndrome; BMI, body mass index; AMH, anti-Müllarian hormone.

Supplementary Table 3 Summary of Calculation Formulae

| Parameter | Formula |
| --- | --- |
| ovarian volume | long diameter (cm) × width diameter (cm) × anteroposterior diameter (cm) × 0.523 |
| BMI | weight (kilograms) / (height (meters) × height (meters)) |
| WHR | waist circumference (cm) / hip circumference (cm) |
| FAI | (TT (ng/mL) ×3.47/ SHBG (nmol/L)) × 100% |
| HOMA-IR | (fasting insulin [uIU/mL] × fasting blood glucose [mmol/L]) / 22.5 |

BMI, body mass index; WHR, waist to hip ratio; FAI, free androgen index; TT, total testosterone; HOMA-IR, homeostasis model of assessment-insulin resistance.
